# Prognostic Value of Various Diagnostic Methods for Long-Term Outcome of Newborns After Hypoxic-Ischemic Encephalopathy Treated With Hypothermia

**DOI:** 10.3389/fped.2022.856615

**Published:** 2022-04-07

**Authors:** Anja Troha Gergeli, Andreja Škofljanec, David Neubauer, Darja Paro Panjan, Jana Kodrič, Damjan Osredkar

**Affiliations:** ^1^Department of Child, Adolescent and Developmental Neurology, University Medical Centre Ljubljana, Ljubljana, Slovenia; ^2^Pediatric Intensive Care, University Medical Centre Ljubljana, Ljubljana, Slovenia; ^3^Health Institution Zdravje, Ljubljana, Slovenia; ^4^Center for Developmental Neuroscience, Faculty of Medicine, University of Ljubljana, Ljubljana, Slovenia; ^5^Department of Neonatology, University Medical Centre Ljubljana, Ljubljana, Slovenia; ^6^Unit of Child Psychiatry of the University Children's Hospital, University Medical Centre Ljubljana, Ljubljana, Slovenia

**Keywords:** hypoxic-ischemic encephalopathy, perinatal asphyxia, hypothermia treatment, long-term neurodevelopmental outcomes, prognostic value

## Abstract

**Introduction:**

Prediction of outcome in newborns with hypoxic-ischemic encephalopathy (HIE) has been modulated by hypothermia treatment (HT). We assessed the predictive value of diagnostic methods commonly used in neonates with HIE for short-term neurodevelopmental outcome and long-term neurological outcome.

**Materials and Methods:**

This longitudinal cohort study followed up 50 term newborns who underwent HT after HIE between July 2006 and August 2015, until preschool age. We estimated sensitivity and specificity for short-term neurodevelopmental outcome at 18 months and long-term neurological outcome at five years based on Amiel-Tison Neurological Assessment (ATNA), electroencephalography (EEG), and magnetic resonance imaging (MRI) performed in the neonatal period.

**Results:**

The accuracy of all neonatal methods tested was higher for long-term neurological outcome compared to the predictive accuracy for short-term neurodevelopmental outcome at 18–24 months. Sensitivity and specificity in predicting unfavorable long-term neurological outcome were: MRI (sensitivity 1.0 [95%CI 0.96–1.0]; specificity 0.91 [95%CI 0.86–1.0]), EEG (sensitivity 0.94 [95%CI 0.71–1.0]; specificity 1.0 [95% CI 0.89–1.0]), and ATNA (sensitivity 0.94 [95%CI 0.71–1.0]; specificity 0.91 [95%CI 0.76–0.98]).

**Conclusion:**

MRI is a powerful predictor of long-term neurological outcome when performed in the first week after HIE in HT treated infants, as are EEG and ATNA performed in the second or third week postnatally.

## Introduction

In high-income countries, moderate or severe perinatal hypoxic-ischemic encephalopathy (HIE) occurs in ~1.6–3/1,000 live births at or near term (1, 2). It is recognized as a major cause of long-term neurologic disability in children (3, 4). Hypothermia treatment (HT) for HIE has been an important advance in routine clinical care of these patients. Several studies have shown the association between HT and improved developmental outcome (DO) at 18–24 months of age (5–7) and few between HT and improved long-term neurological outcome (LTNO) at preschool and school age (8, 9).

Reliable prognostic markers are essential for further treatment improvement, including the use of future neuroprotective strategies and parental counseling (10). In addition, accurate prognosis allows clinicians to intervene early, tailor management, and provide a personalized follow-up plan that could help the child reach his or her full potential. Several routinely used diagnostic methods have been studied in the neonatal period as potential biomarkers of cerebral damage and neurodevelopmental outcomes in children with HT. Amplitude-integrated electroencephalography (aEEG), standard video-electroencephalography (EEG), and magnetic resonance imaging (MRI) have been reported to have good predictive value (11–16). Nevertheless, the predictive values and ideal timing of diagnostic methods have changed in the cooling era and cannot be directly compared with pre-cooling data (14, 17).

The aim of our study was to evaluate and compare MRI, EEG, and Amiel-Tison Neurological Assessment (ATNA) used in the neonatal period in HT treated newborns after HIE to predict unfavorable short-term neurodevelopmental outcome (STNDO) and LTNO in these infants.

## Materials and Methods

This longitudinal cohort study included term newborns born at ≥ 36 weeks' gestation who were treated for moderate or severe perinatal HIE with HT in the Department of Pediatric Intensive Care, University Medical Centre Ljubljana, Slovenia, from July 2006 to August 2015. We conducted a follow-up of the children in the period 2007–2020. Before starting the study, we obtained ethical approval from the National Medical Ethics Committee of the Republic of Slovenia (No. 135/04/12). All the parents consented to participate in the study.

### Participants

Newborns with moderate or severe perinatal HIE, treated with HT according to the TOBY protocol (18), were consecutively enrolled in the study. During the study period, the HT and follow-up protocols remained unmodified. The exclusion criteria were: age older than six hours at the start of HT; the presence of significant congenital anomalies or genetic syndromes; signs of infection/sepsis in the first four days; change in HT protocol for any reason.

### Neonatal Diagnostic Tests

#### Magnetic Resonance Imaging

Magnetic resonance imaging (MRI) was performed in the first two weeks after the sentinel event, median day six, on Siemens 1.5-T Avanto or 3.0-T Trio (Siemens Medical, Erlangen, Germany) scanners. The standard MRI protocol included axial T1-weighted images or inversion recovery-weighted images, T2-weighted images, and diffusion-weighted images. The custom diffusion sequence consisted of 2 × 2 × 2 mm voxels; 9,300 ms repetition time; 96 ms echo time; 1,710 Hz/Px; and 2 b-values, 0 and 1,000. Two reviewers, neonatologist and child neurologist, blinded to the patient outcomes reviewed all MRI and categorized all images according to the Rutherford scoring system (19, 20). Both reviewers received a training course on Rutherford MRI scoring for HIE, provided by a foreign neonatologist, who was very experienced in the technique due to cooperation with prof. M. Rutherford on MRI classification. Interobserver variability was estimated, and a consensus was reached with a third blinded reviewer (the provider of the Rutherford MRI scoring course) in case of disagreement. The Rutherford classification system consists of analyzing four different anatomical regions, namely basal ganglia and thalamus (BGT), posterior limb of the internal capsule (PLIC), white matter (WM) and cerebral cortex. According to the extension of the injury each region is classified from normal, mild, moderate to severely abnormal or in the case of myelination of the PLIC from normal, equivocal to abnormal. We categorized the children into four groups concerning MRI: normal (normal BGT, normal PLIC, normal WM and normal cerebral cortex); mildly abnormal (mildly abnormal BGT, and/or equivocal PLIC and/or mildly abnormal WM and/or mildly abnormal cortex); moderately abnormal (moderately abnormal BGT and equivocal PLIC and/or moderately abnormal WM, and/or moderately abnormal cortex); severely abnormal (severely abnormal BGT and/or abnormal PLIC and/or severely abnormal WM and/or severely abnormal cortex) (Supplementary Figures 1–5).

#### Electroencephalography

Electroencephalography (EEG) was recorded at two weeks of age for at least 60 min with a NicOne EEG amplifier (sampling frequency of 256 Hz; Natus, USA) and EEG caps (sintered Ag/AgCl electrodes; Waveguard, ANT-Neuro, Germany) with 10 electrodes positioned as per the international standard, including a recording reference at the midline. A Grey-Walter montage was generated for annotation according to the standard longitudinal bipolar layout. Two independent neurologists with decades of expertise in neonatal EEG, blinded to patient identity and MRI results reviewed the EEG results for each newborn. When their evaluation differed, they reviewed the EEG together and reached a consensus. We categorized the children into four groups for EEG activity as follows [modified from Shellhaas et al. (21)]: normal—normal background, no epileptiform activity; mildly abnormal—depression of background activity and/or interictal unifocal/interictal multifocal activity; moderately abnormal—low voltage undifferentiated/excessive discontinuity and/or ictal unifocal or multifocal activity; severely abnormal: burst suppression/no activity and/or status epilepticus.

#### Neurological Assessment

An experienced neonatologist examined all the newborns using the standardized Amiel-Tison Neurological Assessment from Birth to 6 Years (ATNA) before discharge from the neonatal unit. The assessment in the neonatal period covers cranial characteristics, namely head circumference growth and potential overlapping of the cranial sutures, alertness, behavior and spontaneous activity, the passive and active tone in limbs and axis, and primary reflexes (22, 23). Patients were categorized into one of four groups according to the presence of optimal, mildly, moderately or severely abnormal neurological signs in accordance with ATNA classification (24).

### Short-Term Neurodevelopmental and Long-Term Neurological Outcome

At 18 months of age, the Bayley Scales of Infant and Toddler Development, third edition (Bayley-III), was used by an experienced clinical psychologist to assess STNDO (25). Infants who could not be tested with the Bayley-III due to severe neurological sequelae were assigned to the moderately/severely abnormal group.

LTNO was determined by a child neurologist using the ATNA and Gross Motor Function Classification System (GMFCS) at five to six years of age (24). The absence/presence of cerebral palsy (CP) was assessed using the criteria of the Surveillance of Cerebral Palsy in Europe (26), and functional activity was graded according to the GMFCS for CP patients (27). Upon these assessments, the patients were categorized into four groups: optimal—no neurological signs; mildly abnormal—isolated abnormal neuro-motor signs (e.g. hyperreflexia, mild coordination, balance problems) or a CP GMFCS grade of level I, moderately abnormal—CP GMFCS grade of level II–III; severely abnormal—CP GMFCS grade of level IV–V.

Unfavorable STNDO was defined as a Bayley-III score <85 in at least one of the three subscales or death and an unfavorable LTNO as moderately/severely abnormal ATNA, pharmacoresistant epilepsy, CP with GMFCS grade of level II–V or death.

### Statistical Analysis

Continuous variables were presented as averages and standard deviations, and categorical variables as frequencies and percentages. Because of the relatively small cohort, the results of all tests (ATNA, MRI, EEG, Bayley-III) were dichotomized into two categories—normal and mildly abnormal in one and moderately and severely abnormal in the other. The association between various neonatal test results and Bayley-III at 18 months of age and LTNO at preschool age was assessed using the chi-square test or the likelihood ratio test (if the expected frequency in any of the cells was <5).

Accuracy, sensitivity and specificity of each diagnostic test, performed in the neonatal period, to predict STNDO and LTNO were calculated.

The significance level for all statistical tests was set at α = 0.05. We performed analysis using SPSS, version 26, and the free Medcalc application: https://www.medcalc.org/calc/diagnostictest.php.

## Results

### Clinical Characteristics of Study Participants

**Figure 1:**
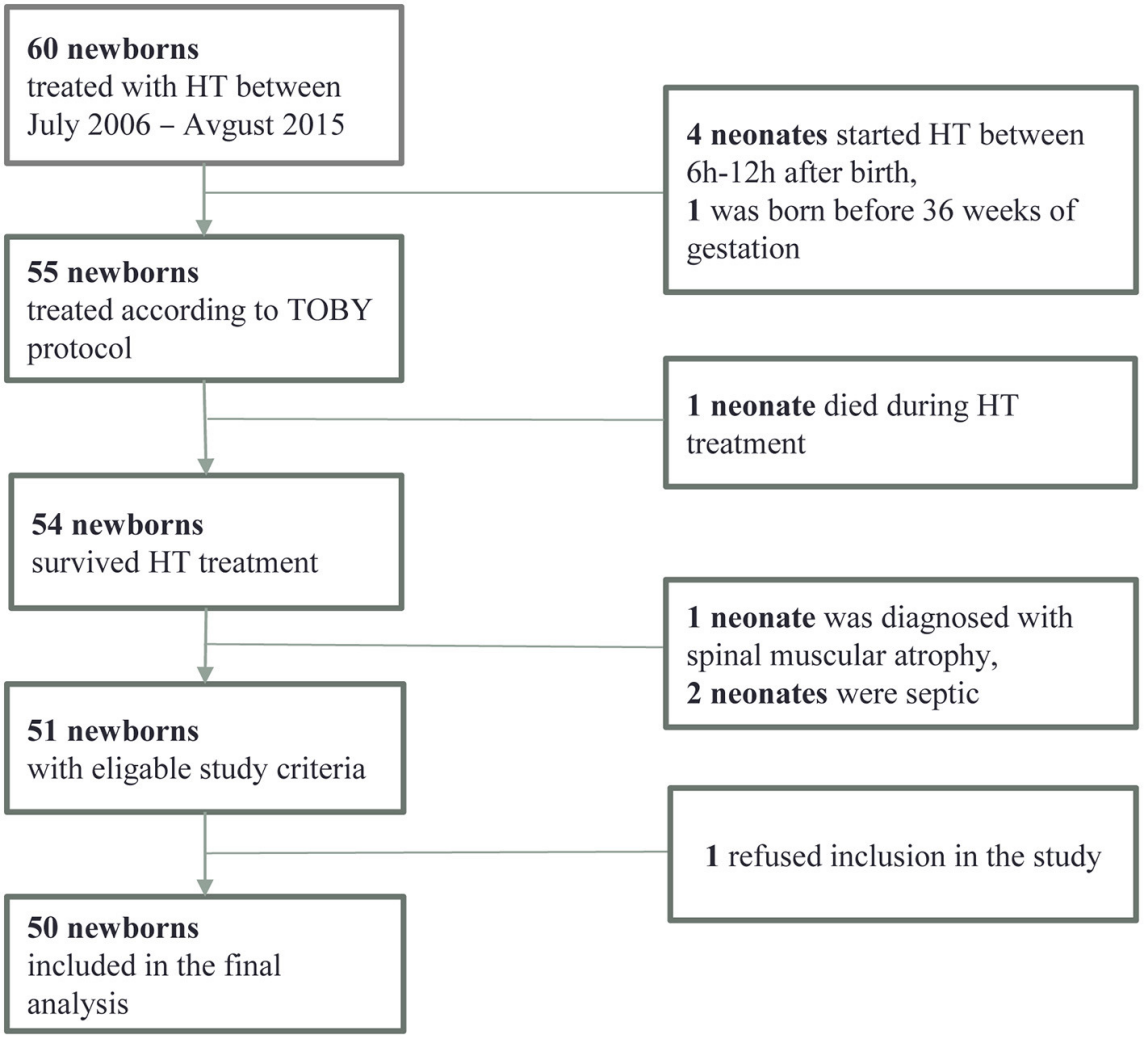
Flowchart of the participants' inclusion process.

Out of 60 HT children, 50 met all the inclusion criteria (shown in Figure 1). The median age at the last follow-up was 5.4 ± 0.52 years.

Clinical characteristics of the children included in the study are summarized in Table 1.

**Table 1:** Clinical data of newborns included in the study.

	***n* = 50**
Birth weight (g)	3,179 ± 526
Gestational age (week)	38.9 ± 1.5
Female sex	24 (48%)
Apgar at 10 min (*n* = 43)	5 (0–9)
Umbilical cord pH at birth (*n* = 38)	6.9 ± 0.2
Sarnat and Sarnat staging for HIE	
Stage II—moderate HIE	21 (42%)
Stage III—severe HIE	29 (58%)

*Values are presented as frequencies and percentages, means ± SD or median (min-max)*.

### Diagnostic Tests in the Newborn Period

MRI, including diffusion-weighted imaging (DWI), was performed in 37/50 (72.0%) infants. In the remaining 14, imaging either was not performed, or the images were lost at follow-up. The median age at MRI was 5.7 (±2.6) days, range 4 to 17 days. Images were categorized as normal in 13/36 (35.1%) infants, mildly abnormal in 11/36 (29.7%), moderately abnormal in one/36 (2.7%), and severely abnormal in 12/36 (32.4%). There were no significant differences in BSITD-III score (*p* = 0.747) or ATNA score in the preschool period (*p* = 0.774) between infants with and without analyzed MRI.

EEG recordings were available for 46/50 (92%) patients. In 4/50 (8%) EEG was not performed. These patients had no observable clinical seizures. The median age at EEG recording (*n* = 46) was 14.3 (± 7.2) days, range 1 to 45 days. EEG was categorized as normal in 15/46 (30.6%) infants, mildly abnormal in 18/46 (36.7%), moderately abnormal in 6/46 (12.2%), and severely abnormal in 10/46 (20.4%).

All newborns were clinically assessed according to ATNA, most commonly at the third week postnatally, before hospital discharge. No infant was categorized in the normal group, while 31/50 (62%) had mildly abnormal neurological signs, 10/50 (20%) moderately abnormal, and 9/50 (18%) had severely abnormal signs.

### Outcome Assessment

We obtained Bayley-III scores in 45/50 (90.0%) children at a median age of 20.0 ± 6.2 months. LTNO, based on ATNA and GMFCS, was acquired in all 50 children at a median age of 5.4 ± 0.5 years. A Bayley-III score <85 in at least one of the three subscales was reported in more than one-third of the patients 17/45 (37.8%). All of these patients were later in the preschool period, when LTNO was assessed, categorized as having moderate three/50 (6.0%) or severe impairment 14/50 (28.0%) according to ATNA and GMFCS scoring. LTNO was categorized as normal in 28/50 (56.0%) and mildly abnormal in five/50 (10.0%) children.

**Figure 2:**
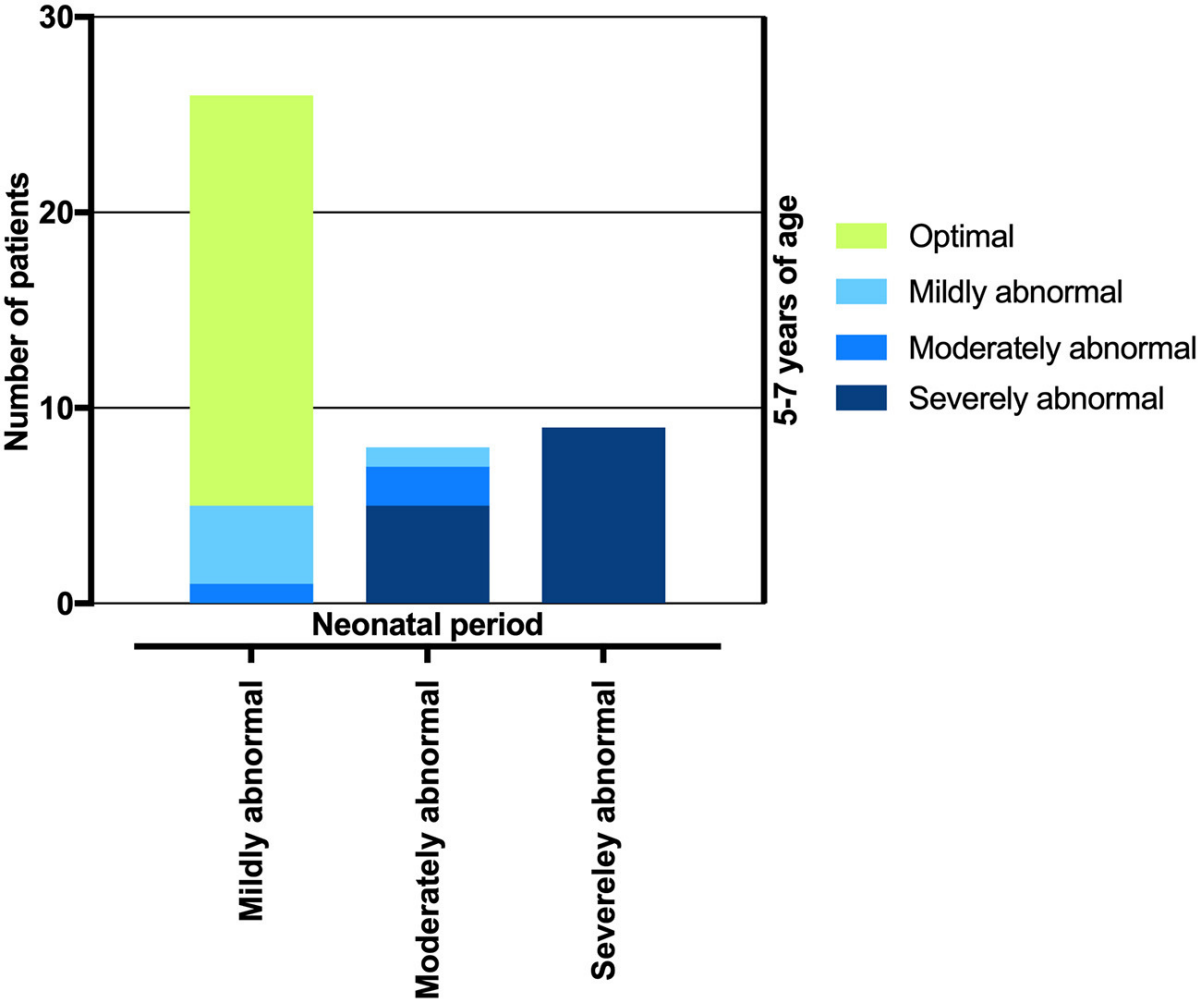
Transition of patients according to neonatal ATNA test result to four different neurological outcome categories according to ATNA and GMFCS assessment in a preschool period.

In the neonatal period, 31/50 (62.0%) newborns had a mildly abnormal ATNA; at the age of five to seven years, 26 (83.9%) of these children had an optimal LTNO, four (12.9%) had a mildly abnormal LTNO, and one (3.2%) had a moderately abnormal LTNO. Ten/50 (20.0%) newborns had a moderately abnormal ATNA; at the age of five to seven years, two (20.0%) of these had an optimal LTNO, one (10.0%) had a mildly abnormal LTNO, two (20.0%) had a moderately abnormal LTNO, and five (50.0%) had a severely abnormal LTNO. All nine/50 (18.0%) newborns with a severely abnormal ATNA had a severely abnormal LTNO at the age of five to seven years (shown in Figure 2).

The vast majority of children (83.9%) with a mildly abnormal ATNA had an optimal outcome at 5.4 ± 0.5 years of age. Therefore, it seemed reasonable to dichotomize the children into two groups according to the ATNA in the neonatal period: optimal/mildly abnormal and moderately/severely abnormal. Similarly, the results of EEG, MRI, Bayley-III score and LTNO in the preschool period were transformed into binary variables (favorable/unfavorable).

A statistically significant association (*p* < 0.001) was found between all dichotomized test scores in the neonatal period and dichotomized STNDO and LTNO.

### Predicting STNDO and LTNO Based on the Results of Diagnostic Tests in the Neonatal Period

**Figure 3:**
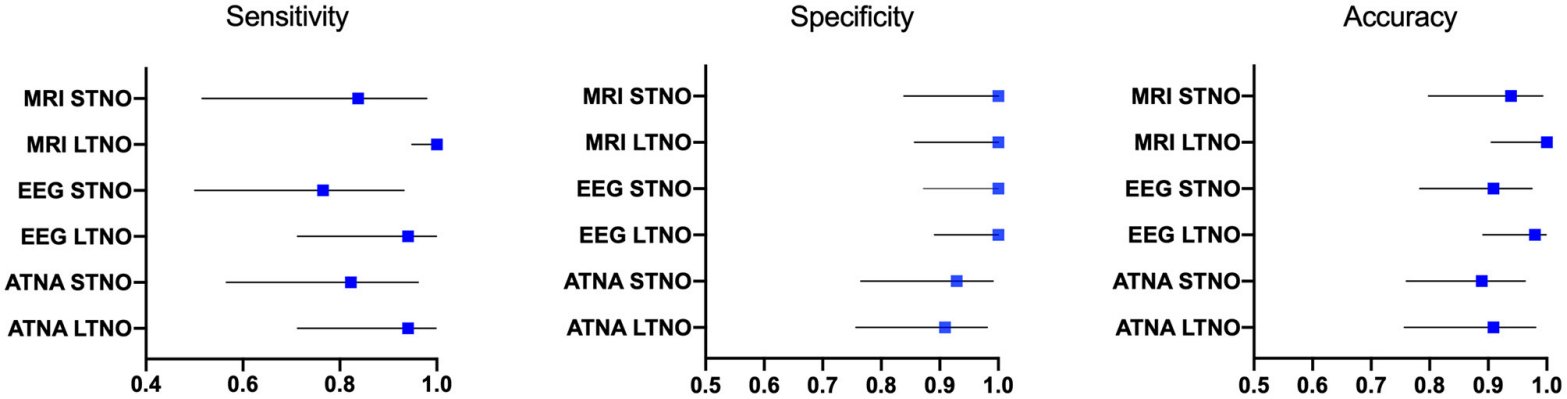
Sensitivity, specificity and predictive accuracy measures with a 95% confident interval of MRI, EEG and ATNA in the neonatal period for unfavorable STNDO based on a Bayley-III and LTNO based on ATNA and GMFCS assessment in the preschool period.

The diagnostic performance of neonatal MRI, EEG and ATNA as predictors of STNDO and LTNO were estimated. MRI showed the highest sensitivity for unfavorable STNDO (0.83, 95% CI 0.52–0.98) and LTNO (1.0, 95% CI 0.96–1.0). Very good sensitivity values for unfavorable LTNO were calculated for EEG (0.94, 95% CI 0.71–1.0), and ATNA (0.94, 95% CI 0.71–1.0) as well. However, for an unfavorable STNDO, the sensitivity of EEG and ATNA were lower (see Figure 3). According to our results, the highest specificity was attributed to MRI [STNDO (1.0, 95% CI 0.84–1.0); LTNO (1.00, 95% CI 0.86–1.0)] and EEG [STNDO (1.0, 95% CI 0.87–1.0); LTNO (1.00, 95% CI 0.89–1.0)]. Estimated overall predictive accuracy was thus highest for MRI in both terms of STNDO (0.93, 95% CI 0.80–0.99) and LTNO 1.0 (95, 95% CI 0.90–1.0), followed by EEG [STNDO (0.90, 95% CI 0.78–0.97); LTNO (0.98, 95% CI: 0.89–1.0)] and ATNA [STNDO (0.89, 95% CI: 0.76–0.96); LTNO (0.92, 95% CI: 0.81–0.98)].

## Discussion

Our study suggests that among the diagnostic methods studied, MRI including DWI was the most accurate predictor of LTNO when performed in the first postnatal week. The predictive accuracy for LTNO exceeded that of STNDO for all methods studied. MRI had good sensitivity (83%) and excellent specificity (100%) for predicting unfavorable STNDO and excellent sensitivity (100%) and specificity (100%) for unfavorable LTNO. When performed in the second or third postnatal week, EEG also proved to be a valuable predictor of LTNO in HT treated newborns. The predictive accuracy for unfavorable LTNO was 98%, with a sensitivity of 94% and a specificity of 100%. In other words, all children with unfavorable EEG results in the neonatal period had unfavorable LTNO, so the estimated specificity was 100%, and of the children with favorable EEG, all but one had favorable LTNO—the estimated sensitivity was 94%. Comparing the three methods in prognostication, neurological assessment based on ATNA seemed to be outperformed by MRI and EEG. However, sensitivity (94.1%) and specificity (90.9%) of ATNA for LTNO were still significant when used in the third week after birth, before hospital discharge. From a clinician's perspective, it is critical not to overlook newborns at high risk for unfavorable outcome in order to provide thorough follow-up to these infants and to engage them in early intervention programs as early as possible. In our study the children with the unfavorable outcome were all identified in the neonatal period based on the moderately/severely abnormal MRI. In view of this, MRI proved to be the most reliable method for predicting unfavorable LTNO, having the highest sensitivity of 100% according to the results of the present study.

Several reports have previously shown that conventional, T1/T2 weighted MRI performed in the first one to three postnatal weeks, is a valuable biomarker of neurodevelopmental outcome in asphyxiated newborns treated with HT (14, 19, 28–31). Diffusion-weighted imaging (DWI) improves the predictive value of MRI in the first postnatal week, when neurological damage in the injured brain is still developing. The typical abnormalities detected by conventional MRI may be missed on T1/T2 weighted images at this age (32). In a meta-analysis by Ouwehand et al. on predictors of outcome after HT following HIE, early MRI (up to day 7) had better predictive value than late MRI (after the first week). Therefore, it was preferred over late MRI, considering that DWI was included in the standardized MRI protocol (10). This is consistent with the results of our study, as the median age at MRI imaging was 5.7 days, and MRI imaging included both DWI and conventional T1/T2 images.

When comparing previous studies on the prognostic value of brain MRI for neurodevelopmental outcome, the heterogeneity of the studies' methodology should be considered. In a recent study, Laptook et al. urged caution in predicting outcome based on neonatal MRI. Their cohort had a low positive predictive value but a high negative predictive value of MRI (33). However, there were significant differences in the methodology of their study. HT was started between 6 and 24 h postnatally (late hypothermia study), different MRI protocols were included in the study, DWI was not mandatory, the median age at MRI acquisition was 7 days (IQR 6–11), and the MRI scoring system used was different. However, our results are consistent with studies of a similar design. Charon et al. determined a sensitivity of 100% for MRI with DWI performed in the first postnatal week for an unfavorable outcome in HT newborns after HIE using the standard HT method. Still, the specificity was 96.3% (34). In the Dutch patient cohort described by Weeke et al. the prognostic values of MRI and DWI were comparable to ours: sensitivity for short-term outcome was 92%, and for long-term outcome 85% and specificity were 95 and 93%, respectively (30).

Multichannel EEG is one of the most important diagnostic tools for assessing the severity of HIE, the presence of seizures, and monitoring improvement over time (35). However, a recent review reported no correlation between EEG/aEEG electrical activity and unfavorable neurodevelopmental outcome in infants with mild HIE (36). A meta-analysis by Liu et al. considered different EEG background activities in predicting death and neurodevelopmental impairment in HT treated infants and reported a pooled sensitivity of 63% and specificity of 82% (37). However, previous studies mostly correlated neurodevelopmental outcome with EEG findings during the first 72 h after birth, whereas the predictive value of EEG after the first 72 h has rarely been studied (10). A delay in the predictive property of EEG beyond the first 36 h has been described in the era of HT (38). It has also been reported that the EEG background pattern at one week of postnatal age can predict the short-term outcome. However, at the age of one month, the prognostic information has usually decreased because the natural tendency of EEG normalization after HIE (14, 39). In the present study, we aimed to determine the value of multichannel EEG at the mean postnatal age of 14 days to minimize the effects of drugs used during HT. We confirmed the valuable prognostic accuracy of EEG for unfavorable LTNO when performed in this time frame, after patient stabilization.

Neurological examination is a readily available, non-invasive, and inexpensive method of assessing infants with HIE. Surprisingly few studies have addressed the issue of predictive validity of the standardized neurological examination in HT treated newborns. Murray et al. reported good predictive value of neurological examination at discharge (positive and negative predictive values were 86 and 72%, respectively), but the results were related to the period before HT (40). Cooled neonates tend to receive sedative, analgesic, and relaxant medications for longer periods than newborns not treated with HT to minimize the stress associated with HT treatment. For this reason, it has been suggested that a standardized neurologic examination be performed at discharge or at least as late as the second or third postnatal week to identify newborns at high risk for neurodevelopmental disorders (14, 16, 41). Specific neurological signs assessed by ATNA have been shown to have good predictive value for STNDO, LTNO, and cognitive problems in preschool and school age. In addition, the ATNA proved to have excellent inter-examiner reliability and represents one of the few standardized neurological assessments that can be used from birth to six years of age (42–45). The results of this study suggest good sensitivity (82.3%) and specificity (92.9%) of ATNA in the neonatal period for detecting children with unfavorable STNDO after HT treatment and even better sensitivity (94.1%) and specificity (90.9%) for unfavorable LTNO. To the best of our knowledge, ATNA has not been previously reported as a valuable prognostic tool in cooled children. One possible explanation may be that neurological examination has been relatively neglected in the last two decades as other technologically advanced methods gained popularity and attracted more research interest. In addition, relevant clinical data, including the neurological examination, are often missing from medical records, which could be a handicap in retrospective studies (14). Again, neurological assessment could be of great value in underprivileged countries where more sophisticated diagnostic methods are less readily available. However, this should be further investigated because it has already been shown that even HT treatment may not have the desired beneficial effect (46).

Accurate prediction of outcome remains a major challenge for clinicians treating newborns with HIE. Currently, several diagnostic methods are used in the neonatal period to assess the extent of brain damage, plan further interventions, and inform parents of their child's likely neurological outcome. The introduction of HT has had a significant impact on outcome, necessitating reexamination of the prognostic value of known methods. The predictive accuracy of the methods used in our study was surprisingly high, possibly suggesting that HT treatment increases the contrast between infants with favorable and unfavorable outcome, whereas fewer infants have intermediate outcome. Despite the impression that infants with mild to moderate injuries benefit most from HT treatment HT could still improve outcome in severely injured newborns, but not enough to change outcome from moderately or severely abnormal to mildly abnormal or normal.

### Prognostic Algorithm

A systematic review highlighted that most studies of prognostic tests were based on small case series or retrospective data or evaluated short-term follow-up (18 months) in infants with HIE before HT. Given the heterogeneity of the tests and outcomes studied, the need for a well-designed and prospective study to test the joint accuracy of multiple complementary tests in prognosis has been emphasized (11). Recently, it has been shown that the combination of different diagnostic modalities in other neonatal risk populations leads to highly accurate prediction of outcomes (10, 11, 47). Unfortunately, we have not been able to establish a predictive model for LTNO using different clinical modalities in the neonatal period, mainly because of an inadequate sample size. According to Agresti's rule of thumb, for each predictor variable included in the logistic regression model, there should be at least 10 events in the smallest outcome categories (48). This means that the regression analysis in our study could consist of only one predictor. The results of our study strongly support the need for large, multicenter, prospective studies focusing on the combination of different clinical modalities in HT treated children to confirm/improve their predictive accuracy.

### Strengths and Weaknesses

We extended follow-up to preschool age to gain insight into LTNO, in contrast to most previously published studies in this area, in which participants were followed only during the first two years of life. In addition, multiple diagnostic tools were evaluated and compared side-by-side on the same population for prognostication, as opposed to evaluating a single method. Of note, the methodology of this prospectively designed study was consistent and did not change throughout the study.

However, the major limitation is the small sample size and missing data. Consequently, we could not perform advanced statistical methods such as multivariate logistic regression analysis, which would likely have improved the quality of the results. The LTNO was based on the ATNA and the GMFCS motor function test. It did not include standardized cognitive assessment tests for detailed assessment of language and cognition. Because of the dichotomization of the LTNO into favorable and unfavorable, we assumed that the parental report and the ATNA, which has been shown to be well-predictive of late-onset cognitive problems, were sufficient (42, 43).

## Conclusion

Our study suggests that MRI was the most powerful tool for predicting unfavorable LTNO when performed in the first postnatal week. EEG and ATNA performed in the second or third week after birth also had significant predictive value.

## Data Availability Statement

The raw data supporting the conclusions of this article will be made available by the authors, without undue reservation.

## Ethics Statement

The studies involving human participants were reviewed and approved by National Medical Ethics Committee of the Republic of Slovenia. Written informed consent to participate in this study was provided by the participants' legal guardian/next of kin.

## Author Contributions

DO, DN, and AT: conceptualization. AT, DO, DN, DP, and JK: methodology and writing–review and editing. AT and DO: data curation, validation, formal analysis, verification, and visualization. AT, AŠ, DN, DP, JK, and DO: investigation. AT: writing–original draft preparation. DO and DN: supervision. All authors contributed to the article and approved the submitted version.

## Conflict of Interest

The authors declare that the research was conducted in the absence of any commercial or financial relationships that could be construed as a potential conflict of interest.

## Publisher's Note

All claims expressed in this article are solely those of the authors and do not necessarily represent those of their affiliated organizations, or those of the publisher, the editors and the reviewers. Any product that may be evaluated in this article, or claim that may be made by its manufacturer, is not guaranteed or endorsed by the publisher.

## Supplementary Material

Supplementary Figure 1 Normal MRI of a term neonate on postnatal day 4 after asphyxia. Classified as normal BGT, normal PLIC, normal WM and normal cortex. T1-, T2- and T1-W (from left to right) images in the transverse plane. Myelination within the PLIC gives a high signal on T1-W and low signal on T2-W images (white arrows). Normal signal intensity of WM on T2-W image (black arrow).

Supplementary Figure 2 Mildly abnormal BGT and equivocal PLIC in a term neonate on postnatal day 4 after asphyxia. T1-, T2- and diffusion-weighted ADC map (from left to right) images in the transverse plane. Hyperintesity on T1-W and hypointesity on T2-W and ADCmap images in ventrolateral thalami (black arrow). Abnormal appearances of PLIC on paired T1- and T2-W sequences (white arrow).

Supplementary Figure 3 Moderately abnormal WM in a term neonate on postnatal day 4 after asphyxia. T1-, T2- and T2-W (from left to right) images in the transverse plane. Diffuse hypointesity on T1 and hyperintesity on T2-W images of WM (black arrows).

Supplementary Figure 4 Moderately abnormal cortex in a term neonate on postnatal day 6 after asphyxia. T1-W images. Increased signal intensity in several anatomical parts, especially in the precentral gyrus (white arrows).

Supplementary Figure 5 Severely abnormal BGT and absence of normal signal of myelination in the PLIC in a term neonate on postnatal day 4 after asphyxia. T1-, T2- and diffusion-weighted ADC map (from left to right) images in the transverse plane. Diffuse hyperintesities on T1-W and hypointesities on T2-W and ADC map images in BGT (black arrow). No visible myelination in the PLIC on paired T1- and T2-W sequences (white arrow).
