# Corrigendum: Prognostic value of various diagnostic methods for long-term outcome of newborns after hypoxic-ischemic encephalopathy treated with hypothermia

**DOI:** 10.3389/fped.2023.1226835

**Published:** 2023-06-22

**Authors:** Anja Troha Gergeli, Andreja Škofljanec, David Neubauer, Darja Paro Panjan, Jana Kodrič, Damjan Osredkar

**Affiliations:** ^1^ Department of Child, Adolescent and Developmental Neurology, University Medical Centre Ljubljana, Ljubljana, Slovenia; ^2^ Pediatric Intensive Care, University Medical Centre Ljubljana, Ljubljana, Slovenia; ^3^ Health Institution Zdravje, Ljubljana, Slovenia; ^4^ Center for Developmental Neuroscience, Faculty of Medicine, University of Ljubljana, Ljubljana, Slovenia; ^5^ Department of Neonatology, University Medical Centre Ljubljana, Ljubljana, Slovenia; ^6^ Unit of Child Psychiatry of the University Children's Hospital, University Medical Centre Ljubljana, Ljubljana, Slovenia

**Keywords:** hypoxic-ischemic encephalopathy, perinatal asphyxia, hypothermia treatment, long-term neurodevelopmental outcome, prognostic value

A Corrigendum on Prognostic value of various diagnostic methods for long-term outcome of newborns after hypoxic-ischemic encephalopathy treated with hypothermia By Troha Gergeli A, Škofljanec A, Neubauer D, Paro Panjan D, Kodrič J and Osredkar D. (2022) Front. Pediatr. 10:856615. doi: 10.3389/fped.2022.856615

**Text Correction**

In the published article, there was an error. We failed to mention a professional who considerately helped in our research in Acknowledgements section.

A correction has been made to **Acknowledgements**, Line 401–402. This sentence previously stated:

“We thank Prof. Linda deVries, MD PhD, for her valuable suggestions on the article and Ass. Prof. Vanja Erčulj for the statistical analyses.”

The corrected sentence appears below:

“We thank M. Martinez-Biarge, MD PhD, for sharing her expertise on MRI scan classification with us, Prof. Linda deVries, MD PhD, for her valuable suggestions on the article and Ass. Prof. Vanja Erculj for the statistical analyses.”

The authors apologize for this error and state that this does not change the scientific conclusions of the article in any way. The original article has been updated.

