# Supplementary figures and images for: Prognostic Value of Various Diagnostic Methods for Long-Term Outcome of Newborns After Hypoxic-Ischemic Encephalopathy Treated With Hypothermia
Source: Front Pediatr. 2022 Apr 7;10:856615. doi: 10.3389/fped.2022.856615 (PMC9021608; doi:10.3389/fped.2022.856615)

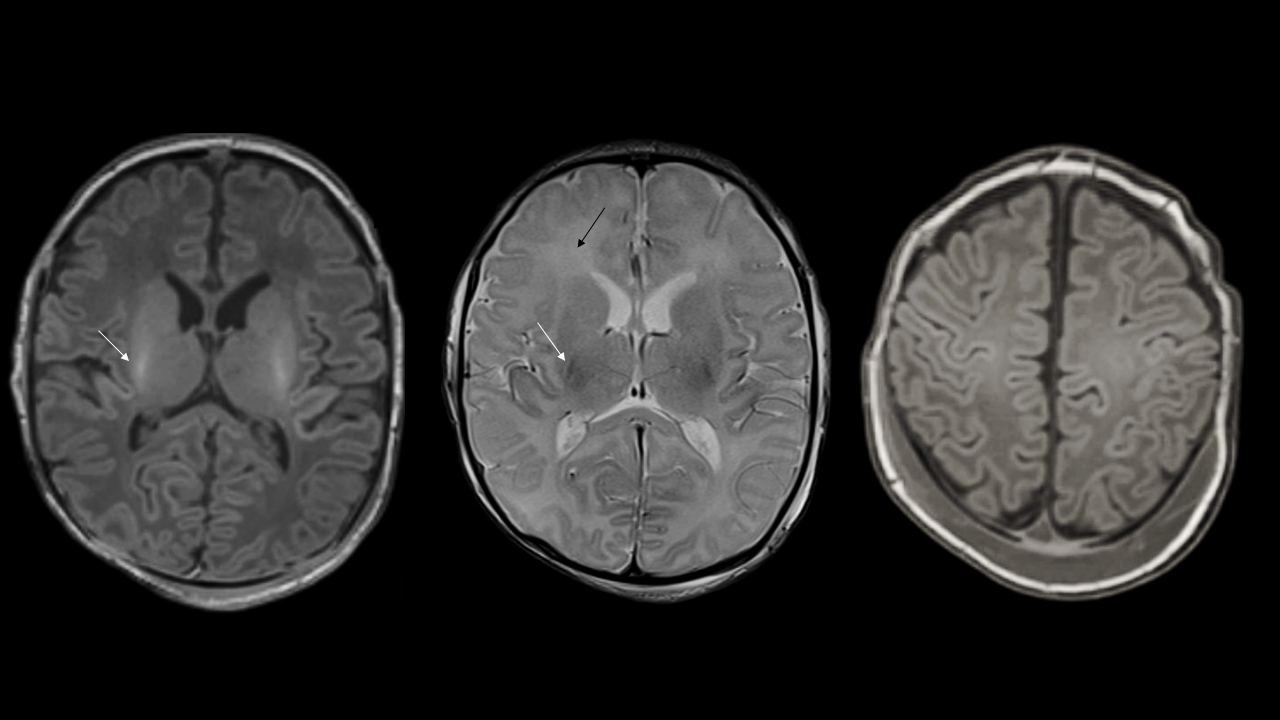

Supplement: Supplementary Figure 1 — Normal MRI of a term neonate on postnatal day 4 after asphyxia. Classified as normal BGT, normal PLIC, normal WM and normal cortex. T1-, T2- and T1-W (from left to right) images in the transverse plane. Myelination within the PLIC gives a high signal on T1-W and low signal on T2-W images (white arrows). Normal signal intensity of WM on T2-W image (black arrow). [file Image_1.TIF]

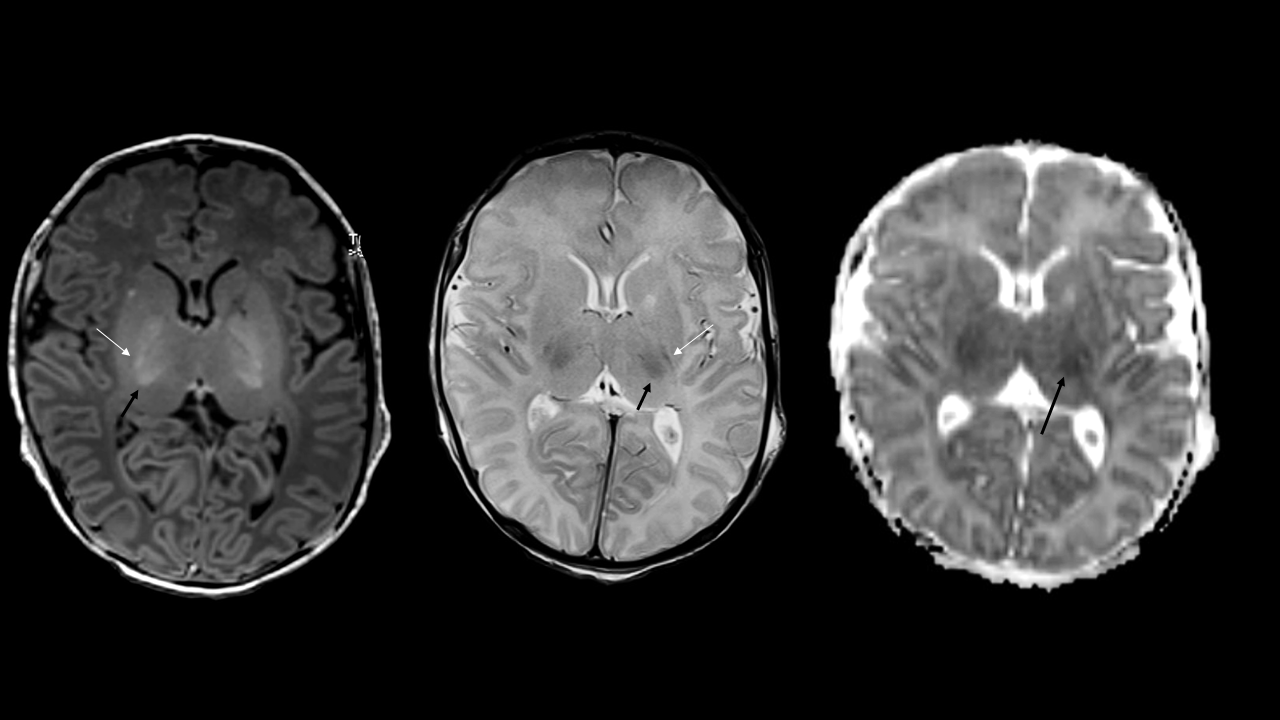

Supplement: Supplementary Figure 2 — Mildly abnormal BGT and equivocal PLIC in a term neonate on postnatal day 4 after asphyxia. T1-, T2- and diffusion-weighted ADC map (from left to right) images in the transverse plane. Hyperintesity on T1-W and hypointesity on T2-W and ADCmap images in ventrolateral thalami (black arrow). Abnormal appearances of PLIC on paired T1- and T2-W sequences (white arrow). [file Image_2.TIF]

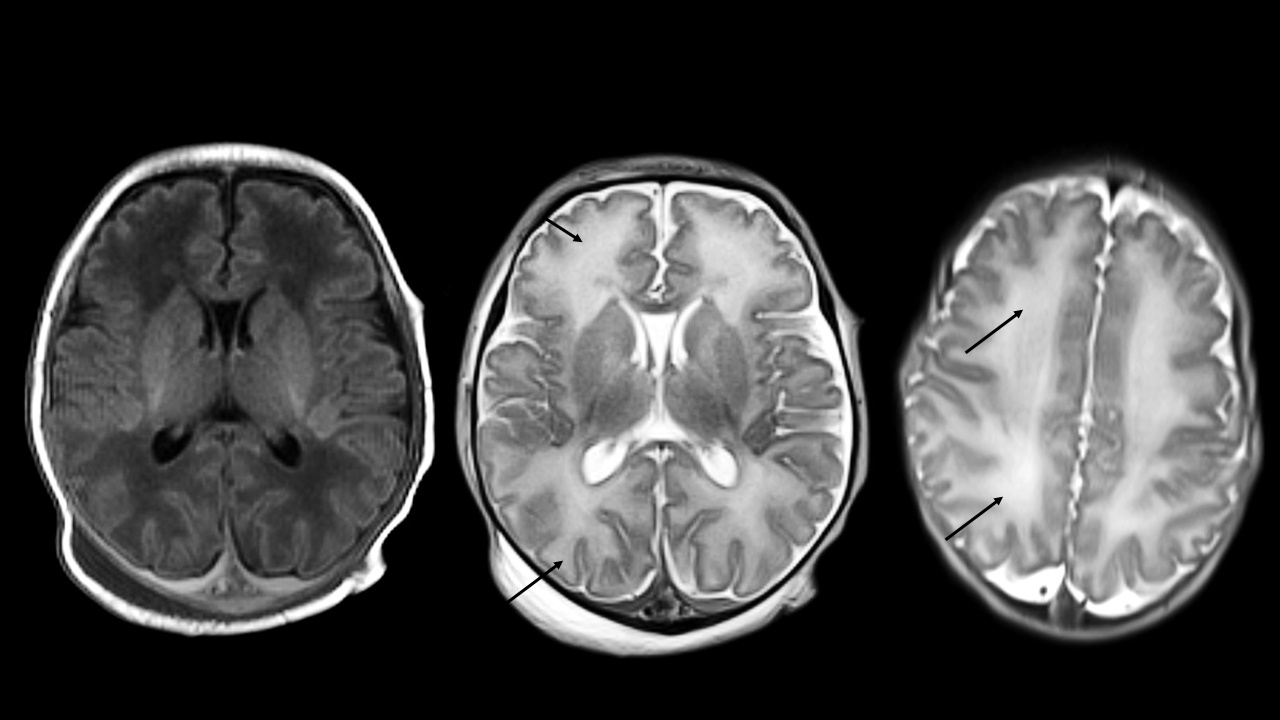

Supplement: Supplementary Figure 3 — Moderately abnormal WM in a term neonate on postnatal day 4 after asphyxia. T1-, T2- and T2-W (from left to right) images in the transverse plane. Diffuse hypointesity on T1 and hyperintesity on T2-W images of WM (black arrows). [file Image_3.TIF]

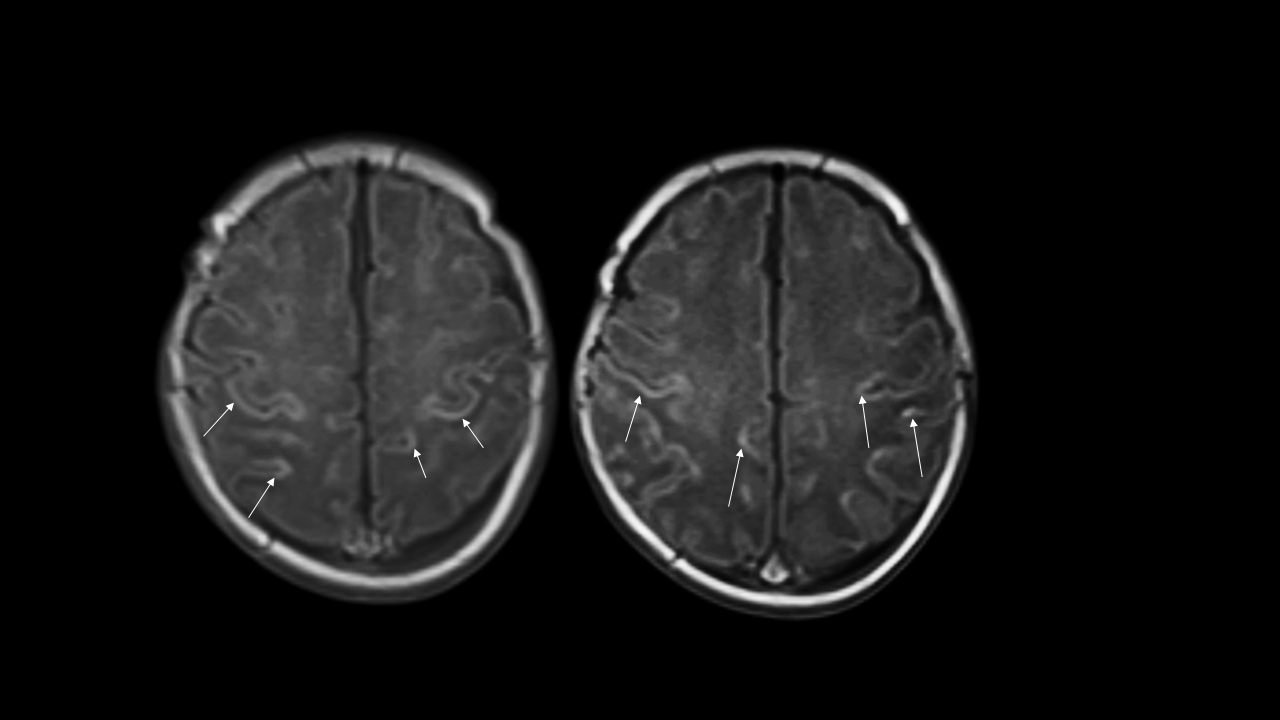

Supplement: Supplementary Figure 4 — Moderately abnormal cortex in a term neonate on postnatal day 6 after asphyxia. T1-W images. Increased signal intensity in several anatomical parts, especially in the precentral gyrus (white arrows). [file Image_4.TIF]

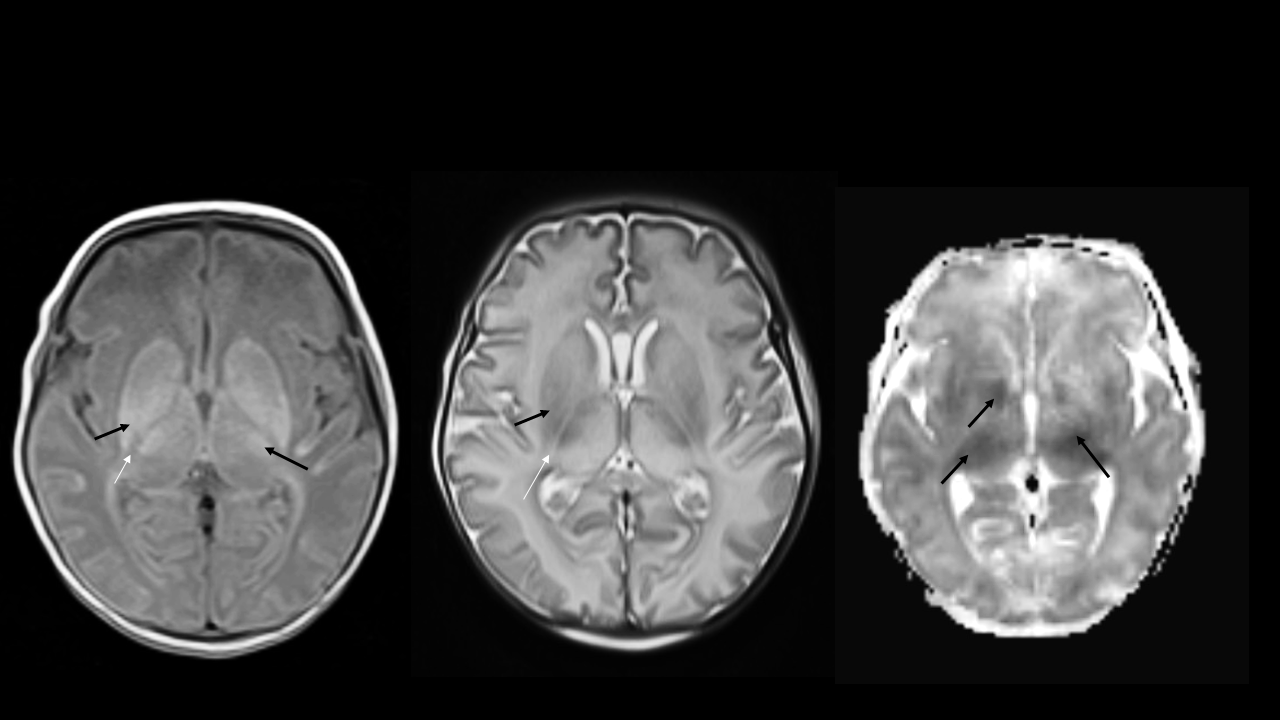

Supplement: Supplementary Figure 5 — Severely abnormal BGT and absence of normal signal of myelination in the PLIC in a term neonate on postnatal day 4 after asphyxia. T1-, T2- and diffusion-weighted ADC map (from left to right) images in the transverse plane. Diffuse hyperintesities on T1-W and hypointesities on T2-W and ADC map images in BGT (black arrow). No visible myelination in the PLIC on paired T1- and T2-W sequences (white arrow). [file Image_5.TIF]
